# Beyond Burden: Cross-Cultural Perspectives on Lay Expertise in Dementia Caregiving

**DOI:** 10.1093/geroni/igaf122.330

**Published:** 2025-12-31

**Authors:** Cameron Beckett

**Affiliations:** University of Chicago, Chicago, Illinois, United States

## Abstract

Alzheimer’s disease and related dementias (ADRD) represent a 21st-century public health crisis, with global prevalence rising alongside aging populations. In the United States, individuals living with Alzheimer’s are projected to double by 2060, intensifying reliance on informal caregivers—mostly family—who navigate complex medical, emotional, and logistical demands without formal training. While existing research documents effects of caregiver burden, including cardiovascular risks, social isolation, and suicidal ideation, this deficit-centric lens obscures caregivers’ potential for adaptation and growth. This multi-sited qualitative study examines how caregivers in Italy and the U.S. acquire “lay expertise” managing ADRD. Drawing on 20 in-depth interviews, ethnographic observations, and grounded-theory analysis, two processes emerge: Building Lay Expertise (practical competence via trial-and-error, self-assessment, and integrating medical advice) and confronting a Constellation of Burdens (role disruption, isolation, and fatigue compounded by structural gaps). Self-efficacy—caregivers’ confidence in managing tasks—buffers strain and sustains skill development. Italian caregivers often view caregiving as a seamless extension of familial obligation, whereas U.S. caregivers experience abrupt, individualized caregiving roles that exacerbate isolation. These differences yield variations in diagnosis timelines and the onset of caregiving burdens. Despite adopting coping strategies—ranging from peer support to creative adaptations—caregivers in both contexts remain at risk of burnout when respite services and community resources are insufficient or poorly tailored. This study reframes caregiving as a process of dynamic skill acquisition and a source of profound vulnerability, underscoring the need for interventions that validate evolving caregiver expertise, and contribute to broader debate on how cultural norms and institutional structures shape family caregiving.

